# The efficacy and safety of modified Gegenqinlian Fomular for advanced colorectal cancer (damp heat accumulation type): A multicenter randomized controlled trial

**DOI:** 10.1097/MD.0000000000027850

**Published:** 2021-12-10

**Authors:** Zhi-Jie Wang, Xiao-Han Wang, Juan Li, Shi-Hang Zheng, Fu-Peng Zhang, Shu-Lan Hao, Xi-Xing Wang, Li-Kun Liu

**Affiliations:** Department of Oncology, Shanxi Province Hospital of Traditional Chinese Medicine, Taiyuan, China.

**Keywords:** colorectal cancer, Gegen Qinlian formula, quality of life

## Abstract

**Introduction:**

CRC, the incidence of the fourth highest among males and the third among females, is one of the malignant tumors that seriously threaten human health. The principle of treatment for advanced stage CRC is a multidisciplinary and comprehensive treatment based on chemotherapy, which always bring significant toxic side effects. CHM has advantages in the treatment of tumors with the effect on improving clinical symptoms and reducing side effects. GGQL formula is mainly used for treating abnormal defecates caused by damp-heat, so we will evaluate the clinical efficacy and safety of modified GGQL formula for patients with advanced CRC with the type of damp-heat in this study.

**Methods:**

Multicenter RCT with two parallel groups in three hospitals planning to recruit 120 CRC patients with the type of damp-heat will be conducted. The control group will be treated by basic antitumor therapy and the treatment group will use modified GGQL formula plus basic antitumor therapy. The primary outcomes will be quality of life, TCM symptom score, PFS and OS, and the secondary outcomes will be performance status, size of tumor, tumor marker in the serum, tumor microenvironment and immune status. All analyses will be based on an intention-to-treat principle. This study was approved by the Human Research Ethics Committee of Shanxi Province Hospital of Traditional Chinese medicine (2021Y-06017). The results will be published in relevant journal.

**Discussion:**

The results of this RCT will contribute to Chinese herbal medicine for treating CRC patients with the type of damp heat accumulation.

**Trial registration:**

ChiCTR2100050754 (September 4, 2021).

## 1 Introduction

Colorectal cancer (CRC) is one of the malignant tumors that seriously threaten human health. According to the 2020 global epidemiological statistics on cancer published by the International Agency for Research on Cancer (IARC) of the World Health Organization, CRC has the second highest mortality rate and the third highest incidence rate of all malignancies with 935,200 deaths and 1,931,600 new cases worldwide. In China, there were about 560,000 new cases of CRC in 2020.^[1]^ The incidence of CRC is the fourth highest among males and the third among females.^[2]^

Patients with CRC have no obvious symptoms in the early stage, only atypical symptoms such as abdominal discomfort, fecal occult blood or indigestion, which are not easy to attract patients’ attention. Once CRC diagnosed, 20% to 30% of patients are already in advanced stage, losing the chance of surgery.^[3]^ The 5-year survival rate for advanced CRC is <10%.^[4]^

Currently, the principle of treatment for early stage CRC is a multidisciplinary and comprehensive treatment based on surgery. While 50% of patients with CRC may have recurrence or metastasis within two years after radical surgery, of which 20% to 30% have local recurrence and 50% to 80% have distant metastasis.^[5]^

For patients who cannot be resected, chemotherapy is recommended.^[6]^ However, chemotherapy drugs often show significant toxic side effects, such as reduction of autoimmunity, inhibition of bone marrow hematopoiesis and gastrointestinal reactions. Therefore, therapies which can increase efficacy and reduce side effects of chemotherapy need to be developed.

Chinese herbal medicine (CHM) has advantages in the treatment of tumors, with the effect on improving clinical symptoms, enhancing patients’ immunity and quality of life, reducing the adverse effects of radiotherapy and chemotherapy, and prolonging survival through the coordinated effects of multiple components, targets and pathways in the process of tumor occurrence and development.^[7]^

In traditional Chinese medicine (TCM) theory, the common pathological factors for CRC are dampness, heat, stasis and deficiency, and dampness and heat can be mutually embedded in the intestine, and some studies have shown that dampness and heat can promote liver metastasis and the mechanism of occurrence may be related to the upregulation of VEGF, MMP-2 and MMP-9 expression.^[8–10]^ Damp-heat is one of the most common types of colorectal cancer. It accounts for about 30% of the overall identification of colorectal cancer.^[11]^

Gegen Qinlian (GGQL) formula is mainly used for treating abnormal defecates caused by damp-heat. Modern medical research has proved that GGQL formula has pharmacological effects such as antipyretic, anti-inflammatory, antibacterial, antiviral antispasmodic inhibiting gastrointestinal motility and enhancing the immunity of the body, and is widely used in the treatment of clinical diseases such as bacillary dysentery, intestinal typhoid, acute and chronic gastroenteritis, and infantile diarrhea.^[12]^ Modified GGQL formula is based on GGQL formula with the addition of Muxiang), Chunpi and Dongguaren, which is clinically effective in patients with intermediate to advanced CRC.

## 2 Objective

In this study, we will evaluate the clinical efficacy and safety of modified GGQL formula for patients with advanced CRC with the type of damp-heat. We will also aim to investigate the relationship between modified GGQL formula and life quality of CRC patients and cellular immunity.

## 3 Methods/design

### 3.1 Trial design

**Figure 1:**
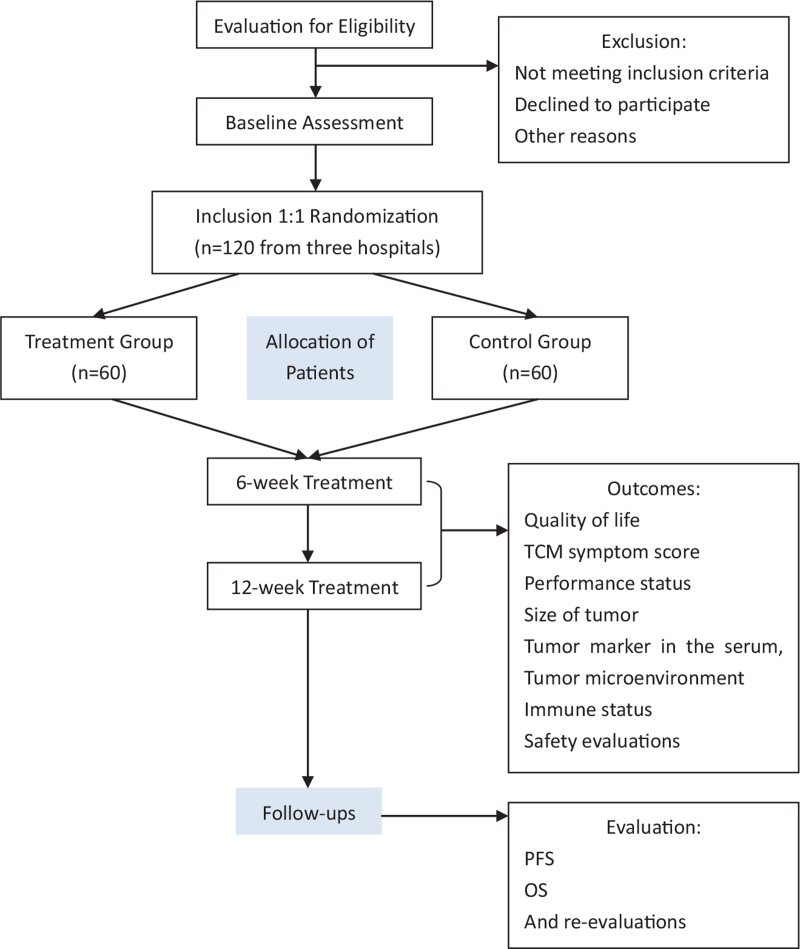
Study flowchart.

This multi-centers, randomized controlled clinical trial with parallel groups of 120 CRC patients with the type of damp-heat will be conducted according to the flow diagram and will last for 2 years, scheduled to start in October 2021 (Fig. 1).

### 3.2 Location and setting

All assessments will be conducted at Shanxi Province Hospital of Traditional Chinese medicine, Shanxi Province Hospital of Oncology, and the Affiliated Hospital of Shanxi University of Chinese Medicine within the department of oncology, Taiyuan, Shanxi, China. And this study was approved by the Human Research Ethics Committee of Shanxi Province Hospital of Traditional Chinese medicine (2021Y-06017).

### 3.3 Participants

#### 3.3.1 Eligibility criteria

The patients are planned to be recruited from the hospitals mentioned above, and the inclusion criteria would be verified.

The inclusion criteria for this RCT are following:

1. Age > 18 years and the excepted survival ≥3 months;
2. Patients with colorectal cancer who have been diagnosed pathologically or cytologically according to the above-mentioned western diagnostic criteria and TCM syndrome type diagnostic criteria;
3. Patients with TNM stage IV, postoperative recurrence, metastasis or loss of operation without operation, did not take Traditional Chinese medicine in recent 1 month;
4. No other primary tumors and serious heart, liver, and kidney diseases;
5. Those who have the telephone number and address of follow-up, and are willing to cooperate with follow-up, have relatively complete medical records, and have signed informed consent.

#### 3.3.2 Exclusion criteria

1. Non-primary colorectal cancer with unclear diagnosis;
2. Unable or unwilling to receive TCM treatment;
3. The patient has developed intestinal obstruction, cannot take medication, needs intravenous high-energy nutrition, or has malabsorption syndrome or other diseases affecting gastrointestinal absorption, or has active peptic ulcer disease.

#### 3.3.3 Discontinuing criteria

Any patients who are unwilling to use modified GGQL formula with or without reasons will quit this study.

### 3.4 Procedures (Template)

CRC patients will be evaluated by an oncologist, and after the initial evaluation, the patient with indication on using modified GGQL formula and fit the eligibility criteria of this RCT will be referred for specific evaluation by an oncologist with at least 3-year experience in the evaluation and prescription of modified GGQL formula. All the included participants would sign terms of free and informed consent.

The participants fitting the criteria of inclusion will be randomized to be divided into two groups: the treatment group (using modified GGQL formula plus basic antitumor therapy) and the control group (basic antitumor therapy).

The first evaluation should occur before treatment and then the participants will accept 12 weeks treatment in the treatment group or the control group. And they will be evaluated each three weeks after interventions.

### 3.5 Randomization and blinding

The process of randomization will be carried out by stratified block randomization method with the block size setting as four and six, and the center was taken as the stratified factor. The participants will be divided into the treatment group and the control group randomly with a ratio of 1:1. We will use the ‘Proc plan’ of SAS9.3 statistical analysis software for generating the random scheme. The central randomization system for clinical research (provided by the Oncology Department of Shanxi Hospital of Traditional Chinese Medicine) will be used for randomization. When qualified patients are enrolled, the randomization personnel or clinical investigators in each center will log into the central randomization system through telephone or Internet to apply for the randomization number.

Because of no herbal medicine used in the control group, it will hardly to blind the doctors and patients involved in this RCT.

### 3.6 Intervention

After specific evaluation by an oncologist, the participant will be divided into one of the two groups according to the randomization.

The treatment group: basic anti-tumor therapy combined with modified GGQL formula.

Basic anti-tumor therapies include:

1. mFOLFOX6 (Oxaliplatin 85 mg/m^2^ d1 + Leucovorin 400 mg/m^2^ d1 + Fluorouracil 400 mg/m^2^ d1 + Fluorouracil 2-2.4 g/m^2^ 46 h), the treatment period is 14 days; or
2. FOLFIRI (Irinotecan + Leucovorin 400 mg/m^2^ d1 + Fluorouracil 400 mg/m^2^ d1 + Fluorouracil 2–2.4 g/m^2^ 46 h), the treatment period is 14 days; or
3. XELOX (Oxaliplatin 85 mg/m^2^ d1 + Capecitabine 850–1000 mg/m^2^ bid d1-14), the treatment period is 21 days.

Modified GGQL formula is mixing and powdering by *Gegen*, *Huangqin*, *Huanglian*, *Muxiang*, *Chunpi*, *Donggua’er*, and *Paojiang*, which offered by Preparation Center of Shanxi Province Hospital of Traditional Chinese Medicine. The patients are planned to be used modified GGQL formula for 12 weeks.

The control group: basic anti-tumor therapy (the same as above).

All patients will be supervised by an experienced oncologist during this study.

### 3.7 Primary outcome measures

1. Quality of life: measured by SF-36 at the baseline, week 6 of treatment, and week 12 of treatment;
2. TCM symptom score: measured by the form of TCM symptom at the baseline, week 6 of treatment, and week 12 of treatment;
3. Progression-free survival (PFS): measured by follow-ups;
4. Overall survival (OS): measured by follow-ups.

### 3.8 Secondary outcome measures

1. Performance status: measured by Karnofsky score at the baseline, week 6 of treatment, and week 12 of treatment;
2. Size of tumor: measured at the baseline, week 6 of treatment, and week 12 of treatment;
3. Tumor marker in the serum: measured at the baseline, week 6 of treatment, and week 12 of treatment;
4. Tumor microenvironment: measured by vascular endothelial growth factor receptor (VEGFR) and Th1/Th2 in the serum at the baseline, week 6 of treatment, and week 12 of treatment;
5. Immune status: measured by subset of the class of T-cells in the serum at the baseline, week 6 of treatment, and week 12 of treatment.

### 3.9 Safety evaluation

All adverse events (AEs) during and after treatment of modified GGQL formula should be recorded detailedly. AEs are including but not limited to nausea, vomiting, changes in stool characteristics and defecation habits, palpitations, headache, dizziness, anorexia, insomnia.

### 3.10 Sample size calculation

The number of participants is capable to detecting according to the results of the previous study (1-year survival rate was 80% and the median overall survival was 25 months in the treatment group; 75% and 22 months respectively in the control group) with an alpha error of 0.05 and beta error of 0.2. Considering the 20% drop-out rates, the sample will be composed of 120 participants randomized into two groups.

### 3.11 Baseline assessments

Sociodemographic data are planned to be collected from the included patients, and also the data on duration of disease and previous herbal medicine treatments.

### 3.12 Statistical analysis

The analysis will follow the principle of intention to treat (ITT). All statistical tests were conducted by two-sided tests and P values less than 0.05 were considered statistically significant. The description of quantitative indicators will calculate the number of cases, mean, standard deviation, median, minimum and maximum. Classification indicators are described by the number of cases and percentages of each category. For quantitative data, a mixed effect model was used to compare the change values of the two groups before and after treatment. The baseline score was taken as a covariable, and the grouping and central effect were considered in this analysis model. The dependent variable is the base change value of comparison point at each time point after treatment. Based on this model, the least square mean (LSMEAN) and the 95% confidence interval of the two groups will be calculated respectively. Kaplan–Meier method will be used to draw OS and PFS survival curves, and log-rank will be used to test the differences between the two groups. The differences of OS and PFS will be discussed in subgroup analysis according to age, sex, left and right colon, gene status, and microsatellite status.

### 3.13 Patient and public involvement

Patients and public involvement are not planned to involve in this RCT.

## 4 Discussion

For CRC patients, the important thing is treating cancer itself, so the importance of optimizing treatment strategies to CRC patients is evident. Quality of life improvement is other object for most CRC patients since it determines the meaning of life of those individuals along with long-term survival benefits.

The results from this RCT will contribute to clinical practice by modified GGQL formula for CRC patients with the type of damp-heat, and guiding future studies on herbal medicine for this subject. Our goal in this study is to accomplish a RCT of high quality that utilizes validated evaluation measures not only for quality of life, effectiveness of treating tumor itself and time of survival in the primary outcomes but also for the tumor microenvironment and immune status of the patients in the secondary outcomes.

### 4.1 Trial status

At the time of submission, recruitment will be carried out. Recruitment will start on October 1, 2021 and is expected to be completed on September 30, 2023. It was registered under on September 11, 2021 and is being financed by National traditional Chinese medicine (TCM) clinical research base (Shanxi Province Hospital of Traditional Chinese Medicine) (JDZXLC-02).

## Author contributions

**Conceptualization:** Shulan Hao.

**Data curation:** Juan Li, Shihang Zheng.

**Methodology:** Fupeng Zhang.

**Software:** Fu-Peng Zhang.

**Supervision:** Zhijie Wang, Likun Liu.

**Writing – original draft:** Zhi-Jie Wang and Xiao-Han Wang

**Writing – original draft:** Zhijie Wang, Xiaohan Wang.

## References

[1] Sung H Ferlayj Siegel RL. Global cancer statistics 2020: GLOBOCAN estimates of incidence and mortality worldwide for 36 cancers in 185 countries. CA Cancer J Clin 2021;71:209–49. 33538338 10.3322/caac.21660

[2] Chen WQ Sun KX Zheng RS. Cancer incidence and mortality in China, 2014. Chin J Cancer Res 2018;30:01–12. 10.21147/j.issn.1000-9604.2018.01.01 PMC5842223 29545714

[3] Li ZY Liang QL Zhou Y. Comparative analysis of clinical epidemiology of 1290 patients with colorectal cancer. J Med Res 2012;41:73–6.

[4] Centelles JJ. General aspects of colorectal cancer. ISRN Oncol 2012;01–19. 10.5402/2012/139268 PMC3504424 23209942

[5] Sun Yan. Internal Oncology. 2001;Beijing: People's Health Publishing House, 623–624.

[6] Chinese Society of Clinical Oncology Guidelines Working Committee. Chinese Society of Clinical Oncology (CSCO) Guidelines for the Treatment of Colorectal Cancer 2019. 2019;Beijing: People's Health Publishing House, 44–76.

[7] Zhao B Du XY. Application of traditional Chinese medicine in integrated treatment of colorectal cancer. Negative 2021;12:06.

[8] Tsuda ON Yasuhide Y Daisuke T. Vascular endothelial growth factor receptor expression as a prognostic marker for survival in colorectal cancer. Jap J Clin Oncol 2009;9:595–600. 10.1093/jjco/hyp066 19535387

[9] Kerbel RS. Tumor angiogenesis. N Engl J Med 2008;358:2039. 18463380 10.1056/NEJMra0706596 PMC4542009

[10] Lu N Ling Y Gao Y. Endostar suppresses invasion through downregulating the expression of matrix metalloproteinase-2/9 in MDA-MB-435 human breast cancer cells. Exp Biol Med 2008;8:1013–20. 10.3181/0801-RM-7 18480415

[11] Chen Z Wang P. Clinical distribution and molecular basis of traditional Chinese medicine ZHENG in cancer. Evid Based Complement Alternat Med 2012;783923. 22829858 10.1155/2012/783923 PMC3398674

[12] Bailey CE Hu CY You YN. Increasing disparities in the age-related incidences of colon and rectal cancers in the United States, 1975-2010. JAMA Surg 2015;150:17. 25372703 10.1001/jamasurg.2014.1756 PMC4666003

